# Magnetic resonance imaging for assessment of cerebrovascular reactivity and its relationship to cognition: a systematic review

**DOI:** 10.1186/s12868-018-0421-4

**Published:** 2018-04-12

**Authors:** Sarah J. Catchlove, Andrew Pipingas, Matthew E. Hughes, Helen Macpherson

**Affiliations:** 1 Centre for Human Psychopharmacology, Swinburne University, Hawthorn, Australia; 2 Centre for Mental Health, Swinburne University, Hawthorn, Australia; 3 Australian National Imaging Facility, St. Lucia, Australia; 4 Institute for Physical Activity and Nutrition, Deakin University, Geelong, Australia

**Keywords:** Cognition, Cerebrovascular reactivity, Vasodilation, Magnetic resonance imaging (MRI), Brain

## Abstract

**Background:**

Cerebrovascular reactivity (CVR) refers to the responsiveness of cerebral vasculature to vasoactive stimuli. CVR is an indicator of brain health and can be assessed using vasodilatory techniques and magnetic resonance imaging (MRI). Using such approaches, some researchers have explored the relationship between CVR and cognition; here we systematically review this work.

**Results:**

We extracted information pertaining to: (1) study location and design, participant characteristics, sample sizes, (2) design of vascular challenge, end-tidal CO_**2**_ (etCO_**2**_) concentrations (if applicable), (3) MRI protocol, (4) cognitive assessment, (5) CVR values, and outcomes of statistical analyses with cognitive tests. Five studies assessed participants with cognitive impairment compared to controls, one studied patients with multiple sclerosis with or without cognitive impairment compared to controls, one examined patients with moyamoya disease with or without cognitive impairment, two investigated patients with Type 2 diabetes mellitus (T2DM), and one was a cross-sectional study with younger and older healthy adults. Cognition was typically probed using the MMSE and tests of executive function, while a number of vasodilatory techniques were employed.

**Conclusion:**

CVR was associated with cognition in six of ten studies, but heterogeneity of study samples, designs and vasodilatory methods may have a role in the inconsistent findings. We make recommendations for future research that includes use of a multi-domain cognitive assessment and standardised hypercapnic challenge with MRI.

## Background

Rising life expectancies, together with declining fertility rates, is leading to rapid global ageing. It is estimated that by the year 2050 the proportion of people aged over 60 years will double from approximately 11 to 22% worldwide [1]. As the population ages, the number of older adults living with impaired cognition and dementia continues to increase. While a variety of mechanisms are thought to contribute to the genesis of cognitive impairment, there is emerging evidence that the signaling between various elements of the neurovascular unit becomes dysfunctional with increasing age, leading to neurovascular uncoupling and dysregulation of cerebral blood flow (CBF) in response to neuronal and metabolic demands [2, 3]. Cerebrovascular reactivity (CVR) refers to the response of cerebral blood vessels to vasoactive stimuli. Dysfunctional CVR impairs blood delivery to brain regions requiring supply, which both precedes and contributes to neuropathology over time. Impaired CVR has been implicated in a wide range of disorders including stroke [4–6], multiple sclerosis [7], hypertension [8], diabetes [9, 10], cardiovascular disease [11] and dementia [12–15]. Further, diminished reactivity has been found to contribute to mild cognitive impairment in the non-clinical general population [16].

This potential link between CVR and cognitive impairment is interesting as it suggests that optimal functioning of the cerebral circulatory system is important for maintaining cognitive functions. The relationship between cognitive decline and numerous vascular anomalies, including stiffness of the peripheral arteries and aorta [17, 18], hypoperfusion [19, 20], cerebrovascular disease [21], and pathology of the carotid arteries [22, 23] has been well established in the literature. To date however, the relationship between CVR and cognitive functions has been poorly understood.

CVR is generally measured as a change in some index of blood flow (e.g., blood flow velocity measured with ultrasound or blood oxygen level dependent (BOLD) signal change measured with fMRI) in response to a vasoactive stimulus. Hypercapnia (increased blood carbon dioxide (CO_2_) concentration) is the most often used stimulus to elicit increased blood flow via vasodilation. Hypercapnia can be induced in several ways including inhalation of CO_2_-enriched air, breath-holding, and rebreathing. While there are numerous vasoactive challenges that can elicit a change in blood flow required for the assessment of CVR, inhalation of CO_2_-enriched air is most suitable due to the practicality of its use and the ease with which it can be standardized [24]. Acetazolamide, a carbonic anhydrase inhibitor, has the same capacity to dilate the cerebral microvasculature via increasing carbonic acid in the arterial blood, and is often used to elicit vasodilation in studies of CVR [25, 26].

Likewise, various tools can be employed to measure the change in blood flow. Most frequently used is the transcranial Doppler ultrasound (TCD). This method is inexpensive, easy to use, non-invasive, is viable for use with large cerebral vessels and has high temporal resolution. However, TCD has low spatial resolution; hence precise regional investigations cannot be performed. Single-photon emission computed tomography (SPECT), positron emission tomography (PET) and other computed tomography (CT)-based technologies also exhibit poor spatial resolution, but are further complicated by the necessity of exposing participants to ionizing radiation. Advances with MRI-based imaging have overcome these limitations whereby CVR assessments can be performed without the use of exogenous contrast agents, and with high spatial resolution so that the responsiveness of blood vessels within discrete brain areas may be studied independently.

Research investigating the relationship between vascular reactivity and cognitive performance has commonly used CT or TCD technology, demonstrating reduced CVR in cognitively impaired patients [12, 14, 27, 28]. Studies using TCD have shown significant relationships between CVR and cognitive status assessed with the mini-mental state examination (MMSE) [28], and with tests of executive function, attention and memory [29]. However, the lack of regional specificity of TCD does not enable an examination of region-specific relationships between CVR and cognitive abilities. To address this apparent gap in the literature, the current work aims to systematically review all research articles investigating the association between cognitive performance and cerebrovascular reactivity to a vasoactive stimulus measured using MRI.

## Methods

### Search criteria

Searches were conducted using Pubmed and Scopus from earliest record until 15th July 2017. Search terms were entered as follows: Pubmed (cognition OR cognitive OR memory OR attention) AND (“cerebral vascular reactivity” OR “cerebrovascular reactivity” OR “cerebral vasoreactivity” OR cvr OR “cerebral vasomotor reactivity” OR “vasomotor responsiveness” OR “cerebrovascular responsiveness”) AND (Humans [Mesh]); and Scopus (TITLE-ABS-KEY (cognition OR cognitive OR memory OR attention) AND TITLE-ABS-KEY (“cerebral vascular reactivity” OR “cerebrovascular reactivity” OR “cerebral vasoreactivity” OR cvr OR “cerebral vasomotor reactivity” OR “vasomotor responsiveness” OR “cerebrovascular responsiveness”)) AND (LIMIT-TO (DOCTYPE, “ar”)).

Only studies published in English using MRI-based CVR assessments and conducted with adult (> 18 years) humans were included. Exclusion criteria included animal studies, CVR assessed with imaging modalities other than MRI, not performing a cognitive/neuropsychological assessment, or not analysing the associations between CVR and cognition. The reference lists of the included studies were also searched.

### Quality assessment and extracted information

Studies deemed eligible were checked for quality using the NIH Quality Assessment Tool for Case/Control Studies and the Tool for Observational Cohort and Cross-Sectional Studies and the PRISMA (Preferred Reporting Items for Systematic reviews and Meta-Analyses) guidelines. Information extracted from the studies related to the country, year of publication, MRI technique and analysis, vasodilatory challenge, CVR values, cognitive/neuropsychological assessment, and participant demographics including age, gender, years of education, cognitive profile and other health status information where available.

## Results

**Fig. 1:**
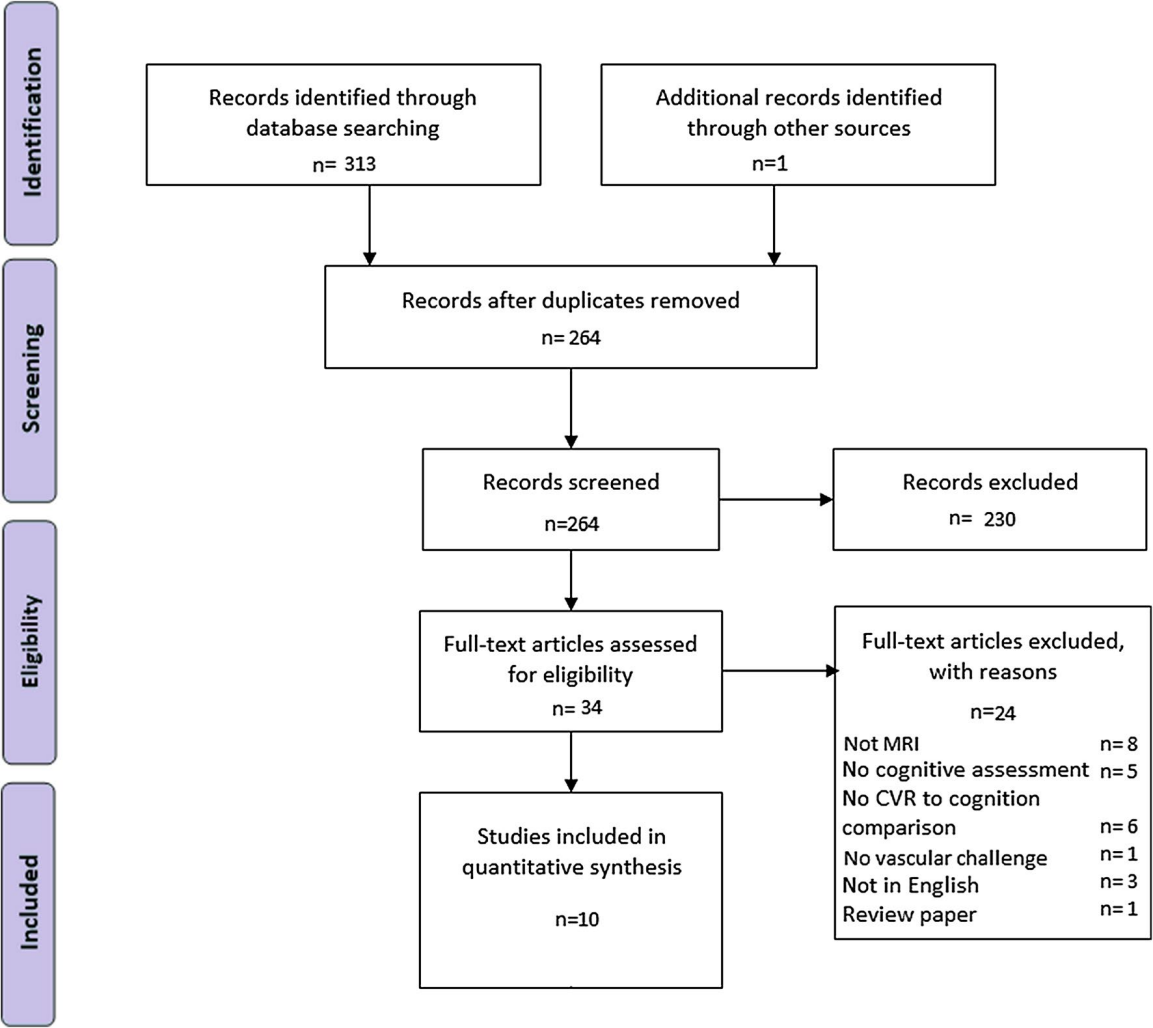
Flowchart of study selection

10 studies were included in the final review [10, 13, 14, 30–36]. All studies were of a fair to good quality as assessed by two independent researchers (SC and HM; See Fig. 1).

### Study demographics and details

Research was conducted in four countries (USA n = 4, Canada n = 2, Switzerland n = 1, France n = 3). Participants in 7 of the studies had an average age of mid-to-late 60’s to early 70’s [10, 13, 30–32, 35, 37], one study investigated adults aged 30–50 years (patients mean age 39 ± 5.91 years, controls mean age 41 ± 6.38 years) [36], one study recruited adults with moyamoya disease aged over 18 years (range 29–73, mean age 40.4 years) [34], while the remaining study involved a cohort of older (mean age 63 ± 5 years) and younger adults (mean age 24 ± 3 years) [33].

Two studies investigated the differences in CVR and cognition between patients with type 2 diabetes mellitus (T2DM) versus healthy controls [10, 32]. Metzger et al. [36] assessed cerebral vasoreactivity and cognitive status in multiple sclerosis patients versus healthy controls. Two studies included patient samples with mild cognitive impairment (MCI) and Alzheimer’s disease (AD) matched with healthy controls [13, 31], while two examined only MCI and healthy controls [35, 37] and one paper examined only AD versus healthy controls [30]. Calviere et al. [34] investigated CVR and cognitive impairment in patients with moyamoya disease. The work by Gauthier et al. [33] was a cross-sectional study assessing differences between groups of healthy younger and older adults.

All studies were single-visit examinations, with the exception of Chung et al. [32] which was longitudinal; participants were assessed at baseline and 2-year follow-up. Table 1 displays patient characteristics.

**Table 1:** Patient characteristics, cognitive assessment and relationship with CVR

Study	Sample (age, mean ± SD)	Cognitive assessment (score ± SD)	CVR in participants	CVR and cognition
Calviere et al. [34]	10 MMD (40.4, 8 female), 6 with DCS	Executive function TMT B Letter and category fluency Stroop interference Brixton test WISC-C WISC-P Attention and processing speed TMT A Coloured dots and words Stroop	CVR < DCS than no DCS	Frontal CVR reduced in cognitively impaired patients, temporoparietal CVR was not different between DCS and no DCS
Cantin et al. [13]	7 MCI (64.1 ± 9.0, 2 female) 9 AD (71.1 ± 6.7, 5 female) 11 HC (65.4 ± 9.3, 6 female)	MMSE MCI 27.4 ± 1.8 AD 21.7 ± 2.2 HC 29.5 ± 0.5 CDR	HC > MCI = AD	CVR correlated with MMSE score in all regions examined
Chung et al. [32]	35 T2DM (65.1 ± 8.0) 30 HC (67.1 ± 10.4) 33 female whole sample 2-year follow-up: 40ppts, 19 T2DM	MMSE (not analysed with CVR) HVLT-R (verbal learning and memory function) ROCF (visual–spatial ability and visual memory function) TMT A & B (executive function) VF (executive function) IADL scale Composite learning and memory T score (average of HVLT-R and ROCF) Composite executive function (average of VF and TMT) T2DM Baseline: 47.5 ± 8.3 2-year follow up: 44.6 ± 10.5 HC baseline: 52.1 ± 7.6 2-year follow up: 56.5 ± 9.9	No significant CVR differences between T2DM and HC at baseline or 2-year follow up	Controls: no significant association between CVR and executive function Decreased CVR associated with decline in executive function in T2DM Regional CVR associated with executive function in frontal and parietal lobes in T2DM
Gauthier et al. [33]	31 younger (24 ± 3, 10 female) 54 older (63 ± 5, 37 female)	MMSE (values not reported) Modified Stroop task (executive function)	Frontal CVR lower in older group, but not significant	Frontal BOLD CVR not associated with Stroop performance
Glodzik et al. [37]	7 MCI (73.4 ± 8.2, 10 female) 17 HC (69.8 ± 6.9, 32 female)	MMSE MCI 27.5 ± 2.4 HC 29.2 ± 1.0 Brief Cognitive Rating Scale GDS	HC > MCI	CVR not related to MMSE, age, or regional brain volumes, in either the entire sample or in HC and MCI subgroups
Metzger et al. [36]	33 MS patients, 12 CI (41 ± 6.27, 7 female), 21 CN (39 ± 5.9114 female) 22 HC (41 ± 6.48, 13 female)	BCcogSEP (short term memory, visual memory, digit spans, working memory, processing speed, go-no-go test, executive function)	Overall MS = HC CI < CN	CVR lower in cognitively impaired patients in whole brain and all regions examined
Richiardi et al. [31]	15 MCI (71 ± 10, 9 female) 20 AD (76 ± 7, 10 female) 28 HC (73 ± 7, 18 female)	MMSE MCI 28 ± 2 AD 25 ± 3 HC 29 ± 1	AD and MCI slower CVR velocity	CVR correlated with MMSE in 10 regions of the DMN
Tchistiakova et al. [10]	18 HTN + T2DM (71.8 ± 5.6, 7 female) 22 HTN only (73.4 ± 6.2), 12 female)	TMT A (processing speed) CVLT (memory) WCST (executive function)	Compared to HTN, HTN + T2DM had decreased CVR in frontal and parietal areas	No significant associations between CVR and cognitive function
Thomas et al. [35]	44 MCI (64 ± 6.6, 26 female) 28 HC (65.6 ± 6.8, 15 female)	MMSE MCI 28.9 ± 1.4 HC 29 ± 1.0 LM (immediate and delayed recall) TMT A & B (executive function) CVLT (memory)	MCI = HC	No significant differences in whole brain grey matter CVR between MCI and HC
Yezhuvath et al. [30]	12 AD (68.7 ± 8.4, 10 female) 13 HC (70.5 ± 8.3, 4 female)	MMSE AD 22.8 ± 4.1 HC 29.6 ± 0.7 CERAD battery CDR BNT (language ability)	HC > AD Compared with controls, AD patients had reduced CVR in rostral brain	CVR in frontal lobe and insula (the primary CVR deficit regions) not related to global cognitive function Significant correlation between CVR and Boston Naming Test score in the frontal and insula regions in AD patients

Mean age and SD in brackets

*MMD* moyamoya disease, *DCS,* dysexecutive cognitive syndrome, *CI* cognitive impairment, *CN* cognitively normal, *HC* healthy controls, *AD* Alzheimer’s disease, *MCI* mild cognitive impairment, *HTN* hypertension, *T2DM* type 2 diabetes mellitus, *CDR* Clinical Dementia Rating, *BCcogSEP* French language test to evaluate cognitive performance in multiple sclerosis, *IADL* instrumental activities of daily living, *ROCF* Rey–Osterrieth Complex Figure, *HVLT-R* Hopkins Verbal Learning Test–Revised, *GDS* Global Deterioration Scale, *LM* logical memory, *TMT A & B* Trail Making Test Parts A and B; *VF* verbal fluency, *CVLT* California Verbal Learning Test, *WCST* Wisconsin Card Sorting Test (-C: categories; -P: perseverations), *CERAD* Consortium to Establish a Registry for Alzheimer’s Disease, *BNT* Boston Naming Test

### Vascular challenge paradigm

Vascular challenges varied between studies. Five elicited hypercapnia via fixed-level CO_2_-enriched gas inhalation. Concentrations varied between 5% CO_2_ in medical air [30, 35], 7% CO_2_ in medical air [31], 7% CO_2_ in 93% oxygen (carbogen) [13] and 8% CO_2_-enriched gas (BACTAL^®^) [36]. Two studies used CO_2_ rebreathing as the hypercapnic manipulation, though both failed to report the length of the rebreathing period, and the size of the reservoir used [32, 37]. Tchistiakova et al. [10] used the breath-hold technique, wherein participants performed a series of 6 × 15 s breath holds following 3 s of exhalation with 30 s of intermittent regular breathing. Gauthier et al. [33] used a computer-controlled gas delivery system which prospectively targeted the partial pressure of expired CO_2_ (etCO_2_) to 40 mmHg for normocapnia, and 45 mmHg for the hypercapnia period, whilst maintaining the expired O_2_ (etO_2_) at 100 mmHg throughout the procedure. The duration of gas delivery also varied across these studies, see Table 2 for details. The remaining study elicited vasodilation via injection of 15 mg/kg acetazolamide [34].

**Table 2:** MR method and parameters, vascular challenge, stimulus effect and regions of interest

Study	MR method	Vascular challenge	Stimulus effect—CVR values	Stimulus effect—etCO_2_ values (mmHg)	Brain regions analysed
Calviere et al. [34]	DSC EPI 1.5T TE: 30 ms	15 mg/kg injection of acetazolamide	Frontal CVR DCS − 13.5 ± 13.2% BOLD/mmHg No DCS 20.2 ± 21.3% BOLD/mmHg Temporoparietal CVR DCS − 7.2 ± 22.2% BOLD/mmHg No DCS 12.7 ± 25.1% BOLD/mmHg	N/A	ROIs: Frontal, temporoparietal
Cantin et al. [13]	BOLD EPI 1.5T TR: 3000 ms TE: 45 ms voxel size 4 × 4 × 4 mm^3^ 32 axial slices	Carbogen (7% CO_2_ in 93% O_2_) Air (1 min) − carbogen (2 min) − air (1 min) × 3, Duration 12 min	MCI 0.36 ± 0.12% BOLD/mmHg AD 0.36 ± 0.13% BOLD/mmHg HC 0.62 ± 0.20% BOLD/mmHg	Baseline MCI 39.9 ± 7.3 AD 34.6 ± 5.6 HC 42.3 ± 5.5 ΔetCO_2_ MCI 13.8 ± 8.9 AD 12.3 ± 5 HC 8.9 ± 3.3	ROIs: frontal, parietal, temporal and occipital lobes, cingulum, insula, striatum and thalamus
Chung et al. [32]	CASL w FAIR 3T Scanning parameters not reported	Vasodilation with CO2 rebreathing, reactivity slope from rebreathing to hyperventilation. CVR values reported are from the vasodilation measure	T2DM Baseline 1.1 ± 0.7 ml/100 g/min 2-year follow up 1.0 ± 1.0 ml/100 g/min HC Baseline 0.69 ± 0.43 ml/100 g/min 2-year follow up 0.39 ± 1.50 ml/100 g/min	Not reported	ROIs: Global, frontal, temporal, parietal, occipital and insula
Gauthier et al. [33]	pCASL w FAIR 3T simultaneous CBF and BOLD BOLD CVR data reported TR: 3000 ms TE1: 10 ms TE2: 30 ms voxel size 4 × 4 mm^2^ 11 slices of 7 mm (1 mm slice gap)	Computer controlled gas delivery system 2 × 2 min blocks of hypercapnia with 2 min of air before and after each block.	Older adults 0.25 ± 0.08%BOLD/mmHg Younger adults 0.25 ± 0.06% BOLD/mmHg	etCO_2_ targeted to 40 mmHg at baseline, 45 mmHg during hypercapnia etO_2_ targeted to 100 mmHg throughout	ROI: frontal lobe
Glodzik et al. [37]	PASL w FAIR 3T TR: 3400 ms TE: 17 ms TI: 1200 ms voxel size 1.2 × 1.2 × 6 mm^3^	CO_2_ rebreathing	MCI Global cortical 0.31 ± 1.2 ml/100 g/min Averaged hippocampus − 0.05 ± 1.7 ml/100 g/min HC Global cortical ± 1.5 ml/100 g/min Averaged hippocampus 1.4 ± 2.7 ml/100 g/min	Baseline MCI 38.7 ± 5.9 HC 39.2 ± 3.1 ΔetCO_2_ MCI 6.2 ± 1.4 HC 7.1 ± 1.8	ROIs: Global cortical, hippocampus
Metzger et al. [36]	BOLD EPI 3T TR:3000 ms TE: 20 ms voxel size 3 × 3 × 3.2 mm^3^ 44 slices	8% CO_2_ enriched-gas (BACTAL^®^) Air (1 min)–BACTAL^®^ (2 min)–air (2 min)–BACTAL^®^ (2 min)–air (2 min)–BACTAL^®^ (2 min)–air (1 min)	Global median CVR Patients CI 0.151 ± 0.06% BOLD/mmHg Patients CN 0.212 ± 0.06% BOLD/mmHg HC 0.207 ± 0.06% BOLD/mmHg	Not monitored, mean etCO_2_ obtained from a standard population	Global median grey matter ROIs: frontal, parietal, occipital, temporal, insula, striatum, thalamus, cingulum
Richiardi et al. [31]	BOLD EPI 3T TR: 2970 ms TEs: 12.3, 29.5, 46.8, and 64 ms voxel size 3.44 × 3.44 × 3.5 mm^3^ 34 axial slices	7% CO_2_ in medical air administered via a nasal canula Air (1 min)–CO_2_ (2 min)–air (2 min)–CO_2_ (2 min)–air (2 min) Duration 9 min	Not reported	Not monitored	Lobe level (7 lobes) and regional level analysis (88 regions)
Tchistiakova et al. [10]	BOLD 3T TR: 2000 ms TE: 30 ms 32 axial slices	Successive breath holds 6 breath holds lasting 15 s each following 3 s expiration period with intermittent 30 s periods of normal breathing CVR calculated as %change in BOLD signal, not corrected for etCO_2_	Not reported	Not monitored	Voxel-wise CVR map Only ROIs that differed between groups specified: left pericalcarine cortex, right inferior parietal, lateral occipital and precuneus, and bilateral cuneus, lingual gyri and superior parietal lobes
Thomas et al. [35]	BOLD 3T Scanning parameters not reported	5% CO_2_ in medical air, administered through a mouthpiece, nose-clip fitted CO2 (1 min)–air (1 min) repeated three times	MCI 0.174 ± 0.04% BOLD/mmHg HC 0.170 ± 0.03% BOLD/mmHg	Not reported	Whole brain grey matter
Yezhuvath et al. [30]	BOLD EPI 3T TR: 3000 ms TE: 30 ms Voxel size 1.7 × 1.7 mm^2^ 25 axial slices	5% CO_2_ in medical air alternating between gas and room air every minute Duration 7 min	Not reported	Baseline AD 33.9 ± 3.5 HC 33.7 ± 5.3 ΔetCO_2_ AD 11.9 ± 1.9 HC 12.2 ± 1.4	Voxel-wise map and ROI analysis ROIs: occipital lobe, temporal lobe, frontal lobe, parietal lobe, insular cortex and subcortical grey matter

*HC* healthy controls, *AD* Alzheimer’s disease, *MCI* mild cognitive impairment, *MMSE* Mini-mental State Examination, *BOLD EPI* blood oxygen-level dependent echo planar imaging, *DSC EPI* dynamic susceptibility contrast-enhanced echo planar imaging, *DCS* dysexecutive cognitive syndrome, *PASL* pulsed arterial spin labeling, *pCASL* pseudo-continuous arterial spin labeling, *T2DM* Type 2 diabetes mellitus, *CI* cognitively impaired, *CN* cognitively normal, *etCO*_*2*_ end-tidal partial pressure of CO_2_, *etO*_*2*_ end-tidal partial pressure of O_2_

### Cognitive/neuropsychological assessment

While the majority of the studies reported more than one cognitive assessment, we were primarily interested in the tests that were analysed in connection with CVR. All but three studies [10, 34, 36] reported mini-mental state examination (MMSE) [38] scores. Of the seven studies that reported MMSE, four investigated the relationship between CVR and MMSE score [13, 30, 31, 37]. Results are reviewed in the discussion section.

Assessments of executive function in connection with CVR were included in five studies. Chung et al. [32] composed a composite measure of the average of verbal fluency and Trail Making Tests A & B scores, Gauthier et al. [33] used a modified Stroop task to measure executive function, and Tchistiakova et al. [10] employed the Wisconsin Card Sorting test (WCST). Calviere et al. [34] used a battery of tests examining executive function (letter and category fluency tests, Trail Making Test B, Stroop interference, Brixton test and a modified version of the WSCT that included both number of categories and number of preservations), and attention (Trail Making Test A, and colored dots and words of the Stroop test) to categorize patients as being cognitively impaired or not. Patients scoring below 5^th^ percentile of the normative mean on 3 or more subtests were considered to have dysexecutive cognitive syndrome (DCS), which defined the cognitively impaired sample in this cohort. Metzger et al. [36] used a similar battery, the BCcogSEP [39] designed to evaluate cognitive impairment in multiple sclerosis. Tasks included assessments of verbal short-term memory, visual perception, digit spans, working memory, processing speed, go-no-go test and verbal fluency. Cognitive status was defined by this evaluation, patients were classified as cognitively impaired if they scored below the 5^th^ percentile of the normative mean of the BCCOG SEP on at least 4 subtests. Other cognitive tasks that did not overlap between studies are outlined in Table 1.

### MRI data acquisition

BOLD fMRI was used in six of the studies [10, 13, 30, 31, 35, 36]. Three papers employed the arterial spin labeling (ASL) MRI technique to measure changes in brain perfusion. Of these, one used pulsed ASL (PASL) [37], one used continuous ASL (CASL) [32] and the final employed pseudo-continuous ASL (pCASL) [33]. However, in the work of Gauthier et al. the images acquired with pCASL sequence were separated into BOLD and CBF time-series data, of which only the BOLD information was used in CVR analysis. Therefore, this work is considered to be a BOLD imaging study. The remaining study used dynamic susceptibility contrast-enhanced (DSC) MRI [34]. All but two studies [13, 34] used an MR scanner with magnetic field strength of 3T. MR protocol information is displayed in Table 2.

### Summary of regional CVR findings

The studies that employed BOLD imaging assessed CVR in various regions-of-interest (ROIs) with some contrasting findings. Results are shown in Table 2. Cantin et al. [13] observed regional impairment in CVR between healthy controls and patients with cognitive impairment, particularly in posterior brain areas, whereas Yezhuvath et al. [30] reported CVR deficits in more rostral regions in patients with AD compared to healthy controls. Cantin et al. [13] investigated CVR in several regions: frontal, parietal, temporal and occipital lobes, the cingulum, the insula, the striatum and the thalamus. Yezhuvath et al. performed a voxel-wise regression and region-of-interest (ROI) analysis using 6 regions: the occipital lobe, temporal lobe, frontal lobe, parietal lobe, insular cortex and subcortical grey matter. This is in contrast to the work by Thomas et al. [35], who found no differences in reactivity between adults with amnestic mild cognitive impairment compared to controls using a voxel-wise comparison of whole-brain grey matter CVR maps. Metzger et al. [36] calculated CVR in 8 regions of interest (ROIs): occipital, parietal, temporal frontal, insula, cingulum, thalamus and striatum, as well as a global median. CVR in all ROIs was significantly reduced in MS patients with cognitive impairment compared to those who were not cognitively impaired.

Another study [31] analysed the BOLD data at the level of overall CVR effect, differences between lobes (7 lobes were delineated as per previous work), brain regions (88 cortical and subcortical regions included), and finally the associations between CVR velocity and the cognitive assessment scores. CVR velocity refers to the temporal dynamics of the CVR response, representing the rate of the vasodilation. It was observed that the largest differences in CVR between AD and healthy controls were seen in the frontal and occipital lobes.

Calviere et al. [34] used 22 ROIs manually drawn on the bilateral frontal and temporoparietal areas of the cerebral cortex, and reference areas in the cerebellum. The mean transit time (MTT) and cerebral blood volume (CBV) values from each area were estimated from perfusion weighted image analysis, and averaged to give one measure from each region. Ratios of CBV in the frontal and temporoparietal areas were calculated relative to the cerebellar CBV, which was used as a control region. The CVR values for each region were estimated from the CBV values relative to the cerebellum. Frontal CVR was lower in cognitively impaired patients with moyamoya disease than those without cognitive impairment.

Gauthier et al. examined CVR using a pseudo-continuous ASL (pcASL) sequence. BOLD data was acquired and intersected with areas of significant signal change in response to the vasoactive stimulus observed with ASL data using cluster analysis to define one frontal ROI [33]. This region was found to be slightly lower in reactivity in older adults compared to younger, yet this difference was not significant, nor was CVR in this region associated with cognitive function.

Chung et al. [32] investigated CVR in the frontal, temporal, parietal, occipital and insula lobes of the brain, as well as calculating a global CVR index. The frontal and parietal lobes were associated with change in executive function in patients with T2DM, but not in healthy controls.

Tchistiakova et al. [10] performed a functional ROI analysis, and reported that there was reduced CVR in several regionals in those with both hypertension and T2DM compared to hypertension alone in the left hemisphere (pericalcarine cortex), right hemisphere (inferior parietal, lateral occipital and precuneus) and the cuneus, lingual gyri and superior parietal lobes bilaterally.

### CVR and neuroimaging correlates of cognitive dysfunction

Seven studies explicitly mentioned correcting for partial volume effects, grey matter atrophy or white matter hyper-intensities (WMH) in their image analyses [10, 13, 30, 31, 33, 36, 37]. In one remaining paper the authors made mention of normalising the perfusion signal for tissue volume, yet did not give further information on the specifics of this procedure [32].

Several studies examined the relationship of CVR to WMH, with some mixed results. Gauthier et al. [33] showed that age, gender and volume of WMH accounted for a significant amount of variance in frontal CVR. Similarly, Yezhuvath et al. [30] found that lower CVR was associated with greater volume of WMH in their cohort of AD and healthy controls. Yet another study investigating the association of grey matter CVR, cardiovascular risk factors and periventricular WMH found that these parameters were intercorrelated [37]. In contrast, Richiardi et al. [31] reported that there was no significant association between severity of WMH and CVR velocity in their cohort of AD, aMCI and healthy controls. Similarly Metzger et al. [36] found that there was no association between CVR and WMH in MS patients, healthy controls or the cohort as a whole.

Of the reviewed papers, only one investigated hippocampal atrophy in relation to reactivity, and it was found to negatively correlate with CVR in the occipital, parietal, striatum and temporal ROIs [13].

### Calculation of CVR

Metzger et al. [36] did not monitor end tidal-CO_2_ (etCO_2_) throughout their experiments, thus they were unable to use this trace as a regressor in their modelling of CVR. This study used mean etCO_2_ obtained from a standard population as a regressor in their general linear model (GLM). Richiardi et al. [31] did not monitor etCO_2_ either. In this work two CO_2_ regression coefficients were calculated analytically to reflect the CVR amplitude and velocity separately, though the authors only report velocity in this paper. These regression coefficients were calculated from mathematical models of nominal and slow etCO_2_ responses to a CO_2_ challenge, to represent the expected responses in healthy subjects and those with slower vessel dilation respectively. CVR velocity was defined in this paper as the rate of vasodilation. While the method of CVR estimation here is acceptable, the unavailability of etCO_2_ data potentially limits the strength of these findings. Similarly, Tchistiakova et al. [10] did not record etCO_2_ throughout the hypercapnic procedure. These researchers calculated CVR as the % change in BOLD signal during 6 × 15 s breath-holds.

Calviere et al. [34] used the regional cerebral blood volume (rCBV) ratio from the regions of interest (ROIs) that was relative to the CBV of the cerebellum (control region). No etCO_2_ was recorded in this study as vasodilation was elicited via injection of acetazolamide, thus the calculation used in this study was: CVR = ([rCBV ratio before acetazolamide − rCBV ratio after acetazolamide]/rCBV ratio before acetazolamide) × 100.

Chung [32] used a rebreathing paradigm to assess vasodilation, vasoconstriction and vasoreactivity separately. Vasodilation was measured as the perfusion increase from baseline during CO_2_ rebreathing normalised to the change in etCO_2_ between baseline and rebreathing. Vasoreactivity was defined as the best-fitting slope between normal breathing, vasodilation and vasoconstriction. It should be noted that the ‘gold standard’ for CVR measurement is more likely the whole vasodilatory range of hypocapnia (elicited by hyperventilation) to hypercapnia [40]. However, CVR is most commonly calculated as the difference in CBF (or surrogate) between baseline and during a vascular challenge divided by the change in etCO_2_ between these conditions, thus the vasodilation measure is taken as CVR, not the vasoreactivity measure in this instance.

The remainder of the studies estimated CVR using the standard calculation:

$${\text{CVR}} = \left( {\left( {{\text{MRIparameter}}_{\text{dil}} {-}{\text{MRIparameter}}_{\text{rest}} } \right)/{\text{MRIparameter}}_{\text{rest}} } \right) \times 100/\Delta {\text{etCO}}_{2}$$

where MRIparameter_dil_ is the CBF or BOLD signal measured during the vasodilated period; MRIparameter_rest_ indicates the CBF or BOLD signal measured at baseline; and ΔetCO_2_ is the difference is end-tidal CO_2_ in mmHg between the two conditions.

### Relationship between CVR and cognition

Of the four papers that analysed the association of CVR to MMSE score, two reported significant positive correlations [13, 31], and two reported no relationship [30, 37]. Metzger’s work found that CVR was lower in MS patients with cognitive impairment compared to non-impaired patients [36], supporting the findings of Calviere et al. [34], who reported that CVR was significantly reduced in patients with moyamoya disease and dysexecutive cognitive syndrome (DCS) compared to patients without DCS. This is in contrast to the results of Thomas’s study, which concluded that whole-brain grey matter CVR was not significantly different between MCI and healthy control groups [35].

Tchistiakova et al.’s [10] research involved three measures of cognitive function, none of which were found to correlate with CVR. These measures were tests of memory, processing speed and executive function. A second study [33] also reported no significant association between executive function as measured by a Stroop task, yet the work by Chung et al. [32] found that CVR decline was linked to a decrease in executive function in T2DM at 2-year follow-up. These results are further discussed below.

## Discussion

This paper systematically reviewed research articles that examined the association between cognition and cerebrovascular reactivity (CVR) using MRI. Six out of ten studies described significant relationships between CVR and cognition, including a longitudinal study which reported that lower CVR was predictive of cognitive decline over a 2-year period. The association of CVR to cognition is more established in individuals with cognitive dysfunctions, while this link is less well-known in cognitively normal adults. There was an over-reliance on imprecise measures of cognition, and the vascular challenges used to measure CVR varied widely.

### CVR is reduced in adults with cognitive dysfunction

CVR was consistently lower in cognitively impaired adults versus healthy controls, or patients without cognitive impairment in the reviewed research (6 of 10 studies). Two studies reported significant correlations between cognition measured by MMSE and CVR in multiple brain regions [13, 31]. These investigations also observed that CVR was significantly reduced in AD and MCI patients, and that AD patients had significantly slower responses to hypercapnia (i.e. CVR velocity was reduced), compared to healthy controls. In contrast, Glodzik et al. and Yezhuvath et al. [30, 37] reported that CVR was not directly related to cognition measured using the MMSE. However, in both of these studies patients with cognitive impairment had lower reactivity than matched healthy controls, seen in the hippocampus in Glodzik et al. [37] and in the prefrontal, anterior cingulate and insular cortices in the study by Yezhuvath et al. [30]. Two other studies reported that CVR was significantly reduced in participants with cognitive impairment compared to those who were cognitively normal [34, 36].

These findings are supported by evidence using other modalities linking dementia severity with cerebrovascular responsiveness [27, 28]. Transcranial Doppler (TCD) ultrasound is often used to measure changes in CBF velocity in investigations of CVR. This method, while temporally precise, lacks spatial resolution, thus its practicality in regional CVR examinations is limited. Nonetheless, research conducted into the relationship between CVR and cognition with TCD has shown interesting results. Silvestrini et al. [28] reported that CVR as measured using the breath-hold index and TCD was the sole predictor of cognitive decline in patients with AD. Moreover, breath-hold index has been found to be associated with early cognitive impairment [41], as well as an increased risk of conversion from MCI to AD [27]. A systematic review of TCD analyses found that CVR to hypercapnia was a good differentiator of dementia sub-types across multiple studies [42]. Overall, the results of the reviewed studies lend support to the hypothesis that CVR and cognitive functioning are linked, evidenced by findings of reduced reactivity in patients with cognitive impairment compared to cognitively healthy controls.

While the data reviewed is suggestive of reduced vascular reactivity in individuals with cognitive impairment, a definitive relationship between CVR and cognition in cognitively healthy adults was not identified. Only one study focused exclusively on cognitively normal adults without chronic health conditions [33], whilst five studies included healthy controls as compared to patients with cognition impairment, and examined CVR and cognition within these participants [13, 30–32, 35]. Two investigations compared patients with cognitive impairment to those without. Metzger studied MS in relation to healthy controls [36], whilst Thomas et al. [34] investigated only individuals with moyamoya disease (MMD). The remaining study investigated CVR and cognition in cognitively normal individuals with hypertension with or without co-morbid T2DM [10]. Within the reviewed studies, imprecise methods were used for evaluation of cognitive function and CVR. Reliance on the mini-mental state exam (MMSE) as the main assessment of cognition in several studies [13, 30, 31, 37] necessitates some caution, as this measure may not be sufficiently sensitive to variation in cognitive capability, nor does it allow for distinction between different cognitive domains [38]. This is evidenced by the findings of Richiardi et al. [30] in which no evidence of a relationship between CVR and cognitive performance was found using measures of global cognitive function, yet a significant correlation was observed with language ability. The MMSE is specifically designed as a screening tool for distinguishing between individuals with and without gross cognitive impairment [38], and as such its usefulness for precise cognitive assessment is not ideal.

Among research that assessed cognitively healthy cohorts and those assessing the cognitive capabilities of individuals with MS or MMD, tests of executive function were used. However the specific tests used to define this construct varied between the five studies, including tasks of inhibitory control, task-switching, verbal fluency, and processing speed, among others [10, 32–34, 36] (see Table 1 for details). Of the two studies assessing patients [34, 36] batteries of neuropsychological tests examining executive function (amongst others) were used to determine cognitive status. While results of two studies showed that executive function was not directly correlated with CVR in either frontal cortex [33] or averaged across the whole brain [10], the two patient studies both reported that CVR was significantly lower in individuals with cognitive impairment compared to those without. This was observed in the frontal region in MMD [34], and in the whole brain grey matter, as well as in a region-of-interest analysis comprising multiple brain areas in MS [36]. Similarly, Chung et al. [32] observed that global CVR was positively associated with executive function in patients with Type 2 diabetes mellitus (T2DM). In T2DM patients, decreased global, frontal and parietal vasodilation at 2-year follow up was linked to accelerated declines in executive function. The executive function task was composed of separate tasks of verbal fluency and Trail Making Task A, which assesses task-switching and visual attention. The studies that failed to observe any association between CVR and executive function used tasks that assessed interference and flexibility in thinking (Stroop and the Wisconsin Card Sorting Task, respectively), whilst the patient studies that did observe an association defined cognitive status on the basis of multiple executive function tasks. Thus it could be that some aspects of executive function are more related to CVR than others. Notably, it is thought that there are from 3 to as many as 7 distinguishable executive abilities [43], hence a more comprehensive approach to assessment would be necessary to draw definitive conclusions.

### Relationship between CVR and cognition may be mediated by cardiovascular risk factors in cognitively healthy adults

There is evidence that CVR is related to executive function in populations with cardiovascular risk [10, 32]. One study [10] reported that CVR was significantly lower in those with comorbid hypertension and T2DM versus participants with hypertension alone. Similarly, Chung et al. [32] reported that higher inflammatory markers in T2DM were linked to greater reductions in CVR, which resulted in accelerated cognitive decline over a 2-year period. Gauthier et al. [33] reported an association between cognitive performance and aortic pulse wave velocity (PWV) in their healthy cohort, yet no direct link between CVR and cognition was observed. This finding was interpreted as indicating that declining vascular health, even in primary stages, negatively impacts cognition. Due to the above-average health of the cohort only minor differences in cerebrovascular properties were seen, as compared to larger changes seen in aortic elasticity between younger and older adults. Small blood vessel changes, coupled with the known low signal to noise ratio (SNR) present in BOLD imaging was posited to explain the unexpected lack of relationship observed between CVR and cognition in this study.

The relationship between cardiovascular risk and CVR was more clearly demonstrated by Glodzik et al. [37], who reported moderate negative correlations between the two in the hippocampus (*r *= − 0.41) and cortical grey matter (*r *= − 0.46) in both patients and healthy controls. Likewise, there is evidence of a link between reduced CVR and increased vascular risk in previous studies using MRI [44] and TCD [45]. Together, these findings may indicate that decreased reactivity may be the result of poor vascular health in general, and this is the primary factor triggering neurocognitive decline. Extensive evidence indicates that risk factors for cardiovascular disease precede and facilitate cognitive deterioration in aging [46–48].

Cardiovascular factors can result in dysfunctional reactivity in specific brain regions, leading to hypoperfusion which may pertain to cognitive impairment. The discrepancies between these three studies are multifaceted including use of different: executive tasks; methods of inducing hypercapnia; and, different imaging techniques [10, 32, 33]. These discrepancies’ limit the generalisability of the findings to a wider cohort; however, it can be seen that there is a possible association between cardiovascular risk factors and CVR which may mediate the relationship between CVR and executive function in cognitively healthy individuals. Further studies are needed to confirm these associations.

Similarly, there is the possibility that the observed relationships between CVR and cognition could be mediated by the presence of other cerebral pathologies known to disrupt cognition, such as white matter hyper-intensities (WHM) and hippocampal atrophy. It is understood that severity of WMH corresponds to cognitive decline [49, 50], and evidence has shown that normal-appearing white matter that progresses to WMH has lower CVR than areas that do not progress [51]. Within the reviewed articles, the relationship of CVR to WMH was mixed, with three [30, 33, 37] of five studies reported a significant correlation. Interestingly, all three of these papers observed significant relationships between cognition and CVR, thus it is apparent that continued research investigating these associations is necessary.

### Methodological considerations

While the results of the reviewed studies are inconsistent, this is likely influenced by heterogeneous samples, imprecise cognitive testing instruments (as outlined above), varying procedures for inducing vasodilation and differences in imaging protocols.

### Differences in vascular challenge

All studies induced an increase in cerebral blood flow; however, not all manipulations are equal in their capacity to elicit vasodilation. Whilst the breath-hold method is used widely, is inexpensive and efficient in inducing CBF changes, this technique may produce less reproducible stimuli and/or data due to participant compliance, as well as individual differences in breath hold capacity. Breath-hold and re-breathing procedures during MR imaging also present potential risk of motion artifacts, which may result in undesirable signal differences [52]. It is well established that the strength and duration of the stimulus effects the cerebrovascular response [53]. Inhalation of CO_2_-enriched gas mixture has been shown to be a more highly reliable means to induce hypercapnia and stimulate the cerebral vasculature [54–56].

Prospective targeting of etCO_2_ has been deemed the most standardisable stimuli for measuring CVR in a recent review paper [24], yet only one study included in the current work employed this technique [33]. It should be noted however, that the literature is far from a consensus on which vascular challenge is most appropriate for assessment of CVR.

Five studies used the more traditional method of inhalation of fixed-level CO_2_-enriched gas. Differences may appear somewhat minor—a discrepancy of 2% CO_2_ concentration between the 7% used by Richiardi et al. [31] and 5% by both Thomas [35] and Yezhuvath et al. [30]; while Richiardi et al. and Cantin et al. [13] used the same concentration of CO_2_ (7%), the latter study mixed the gas with 93% O_2_, a substance known as carbogen, rather than medical air, which is balanced with N_2_. The gas concentration utilised by Metzger [36] was slightly higher again (8% BACTAL^®^), and it should be noted that the composition of this gas mixture was unreported, and unable to be identified from an internet search.

While these differences in CO_2_ concentration may seem trivial, the evidence suggests that the relationship between BOLD signal and PaCO_2_ is non-linear, thus CVR results may be dependent on the CO_2_ concentration used, as well as baseline PaCO_2_ [57]. The use of carbogen (and potentially, BACTAL^®^) as the vasoactive stimulus [13], rather than standard medical air, has implications for the measurement of CVR, particularly when combined with BOLD imaging [54]. The percentage of oxygen present in carbogen is greater than that in the atmosphere, which will result in an increase in arterial partial pressure of O_2_ (PaO_2_), and possible vasoconstriction, confounding the vasodilatory response intended for CVR measurement. By nature, BOLD imaging relies on the ratio of oxygenated to deoxygenated hemoglobin in the blood, and any increase in PaO_2_ in the brain will elicit unwanted changes in the BOLD signal. BOLD is also sensitive to changes in blood flow, volume and oxygen metabolism, whilst ASL measures flow only, and is not affected by changes in blood oxygenation.

Two studies employed CO_2_ rebreathing [32, 37]. Both of these papers lacked information regarding the volume of the respiration reservoirs used and length of rebreathing period in the paradigms, hampering comparability. Chung et al. [52] also failed to report the end-tidal CO_2_ values. The speed at which partial pressure of CO_2_ (PaCO_2_) rises will be affected by respiration rates and the volume of the rebreathing reservoir, ultimately influencing the measured CVR value. Likewise, breath-holding may produce confounding variables, as the rise in PaCO_2_ during breath-holding varies between individuals due to differences in lung size and metabolic rate [24]. This method also relies heavily on participant compliance and may be difficult or uncomfortable for some to perform [58].

An acetazolamide challenge was used in one study [34]. Whilst this method is safe, does not rely on subject cooperation and is widely used in clinical settings, administration via injection is invasive, and a standardized dose may not produce the replicable stimulus necessary for CVR estimation due to individual variability [24]. For the purposes of participant comfort, less invasive stimuli would be preferable for measurement of CVR.

There are multiple options for inducing an increase in cerebral blood flow, however future research in this area would benefit from a more standardized and reproducible approach, particularly if the purpose is a simple measure of cerebrovascular response amplitude. Inhalation of CO_2_-enriched gas is an easily implemented and standardizable method, with fewer contraindications than rebreathing and breath-holding. While computer-controlled etCO_2_ prospective targeting is the most clinically standardizable technique, it requires expensive equipment which is not readily available in most research facilities. At a minimum, researchers should take care to provide sufficient information regarding vascular challenge techniques so that comparisons may be made between studies.

### Variations in imaging protocol

ASL, BOLD and DSC imaging methods are all considered valid for measurement of CVR, yet the results from these are not directly comparable. While MR imaging has a clear advantage of spatial specificity over ultrasound and CT-based methods, all three methods present possible drawbacks in regard to measuring CVR. BOLD, the most commonly used method, acquires images via the complex combination of blood volume, flow and oxygenation metabolism in the brain and thus is affected by subtle variations in any of these parameters, despite not directly measuring blood flow per se. BOLD is also known to be more sensitive to the baseline level of vascular tension than perfusion MRI [59]. ASL, while being more physiologically precise, has limited spatial coverage, lower signal to noise ratio, and is generally considered less sensitive for measures of CVR [33]. As BOLD is more commonly used and also more accessible on conventional MRI scanners, it is the currently preferred sequence for CVR measurements, although rapid development of new ASL pulse sequences enabling global brain coverage may render it the favored method in the future. Both ASL and BOLD MRI have been widely used in studies of hemodynamic function and cognitive performance in both healthy [60, 61] and patient samples [62, 63]. DSC is less commonly employed in CVR measurement studies, most likely due to the necessity of an injection of an exogenous contrast agent. Both BOLD and ASL are non-invasive, well tolerated and easily repeatable, thus either of these methods are considered preferable over DSC MRI for measurement of CVR in research studies.

## Conclusion

The connections observed between hemodynamic dysfunction and cognitive impairment observed in the majority of these studies warrants further investigation. Those affected by cognitive impairment were more likely to exhibit decreased CVR compared to healthy controls, as were individuals with greater cardiovascular risk factors. Previous research using alternative methods have given strong indication of the causal relationship between dysfunctional CVR and cognitive deterioration. Given that vascular risk factors are often modifiable, development of vaso-protective therapies may prevent or slow the progression of cognitive decline.

Due to the fact that there is still so much to investigate regarding which type of vasoactive modulation and imaging protocol provides the richest set of data to assess vascular function, recommendations for measurement of CVR response amplitude include the inhalation of a set concentration of CO_2_-enriched gas, in combination with either ASL or BOLD MRI, provided that the whole brain is imaged. Future research should also employ more comprehensive neuropsychological examination to further unravel the nature of the association between cerebrovascular reactivity and cognition.
